# Analysis of the potential regulatory mechanisms of female and latent genital tuberculosis affecting ovarian reserve function using untargeted metabolomics

**DOI:** 10.1038/s41598-024-60167-7

**Published:** 2024-04-25

**Authors:** Zhimin Wang, Xueyan Zhang, Bai Dai, Debang Li, Xiujuan Chen

**Affiliations:** Reproductive Medicine Center, Affiliated Hospital of Inner Mongolia Medical University, Hohhot, 010050 People’s Republic of China

**Keywords:** Female genital tuberculosis, Latent genital tuberculosis, Ovarian reserve function, Ovarian response, Untargeted metabolomics, Infertility, Urogenital reproductive disorders, Tuberculosis

## Abstract

Female and latent genital tuberculosis (FGTB and LGTB) in young women may lead to infertility by damaging ovarian reserve function, but the regulatory mechanisms remain unclear. In this study, we investigated the effects of FGTB and LGTB on ovarian reserve function and potential regulatory mechanisms by untargeted metabolomics of follicular fluid, aiming to provide insights for the clinical management and treatment approaches for afflicted women. We recruited 19 patients with FGTB, 16 patients with LGTB, and 16 healthy women as a control group. Clinical data analysis revealed that both the FGTB and LGTB groups had significantly lower ovarian reserve marker levels compared to the control group, including lower anti-Müllerian hormone levels (FGTB: 0.82 [0.6, 1.1] μg/L; LGTB: 1.57 [1.3, 1.8] μg/L vs. control: 3.29 [2.9, 3.5] μg/L), reduced antral follicular counts (FGTB: 6 [5.5, 9.5]; LGTB: 10.5 [7, 12.3] vs. control: 17 [14.5, 18]), and fewer retrieved oocytes (FGTB: 3 [2, 5]; LGTB: 8 [4, 8.3] vs. control: 14.5 [11.5, 15.3]). Conversely, these groups exhibited higher ovarian response marker levels, such as longer gonadotropin treatment days (FGTB: 12 [10.5, 12.5]; LGTB: 11 [10.8, 11.3] vs. control: 10 [8.8, 10]) and increased gonadotropin dosage requirements (FGTB: 3300 [3075, 3637.5] U; LGTB: 3037.5 [2700, 3225] U vs. control: 2531.25 [2337.5, 2943.8] U). All comparisons were statistically significant at P < 0.05. The results suggested that FGTB and LGTB have adverse effects on ovarian reserve and response. Untargeted metabolomic analysis identified 92 and 80 differential metabolites in the control vs. FGTB and control vs. LGTB groups, respectively. Pathway enrichment analysis revealed significant alterations in metabolic pathways in the FGTB and LGTB groups compared to the control group (P < 0.05), with specific changes noted in galactose metabolism, biotin metabolism, steroid hormone biosynthesis, and nicotinate and nicotinamide metabolism in the FGTB group, and caffeine metabolism, primary bile acid biosynthesis, steroid hormone biosynthesis, and glycerophospholipid metabolism in the LGTB group. The analysis of metabolic levels has revealed the potential mechanisms by which FGTB and LGTB affect ovarian reserve function, namely through alterations in metabolic pathways. The study emphasizes the importance of comprehending the metabolic alterations associated with FGTB and LGTB, which is of considerable relevance for the clinical management and therapeutic approaches in afflicted women.

## Introduction

Tuberculosis (TB) is a chronic infectious disease caused by *Mycobacterium tuberculosis* (Mtb) infection^1^. TB poses a serious threat to human health and is an important global public health concern. WHO data showed that approximately 10.6 million individuals were infected with TB in 2021, and approximately 1.6 million individuals died due to TB^2^. Countries with high TB incidence rates are mainly in Asia and Africa, but the TB incidence rate is increasing even in developed countries in Europe and North America^2^.

In countries with high TB prevalence, female genital TB (FGTB) is a major cause of infertility. FGTB is usually secondary to pulmonary TB (PTB) or extrapulmonary TB (EPTB) (which is found in the kidneys, meninges, and skeletal and gastrointestinal systems). In global EPTB and PTB cases, FGTB incidence rates are 9–20% and 5–13%, respectively^3,4^. Mtb mainly infects the reproductive tract via the blood-borne and lymphatic route or by direct transmission from abdominal TB but rarely by sexual transmission^5^. FGTB is mostly found in women of childbearing age (20–40 years) and lacks characteristic manifestations, and affected women often seek medical attention because of infertility^6,7^. Approximately 60–80% of the women with FGTB suffer from infertility because FGTB affects the patient's reproductive organs, mainly the fallopian tubes (95–100%), followed by the endometrium (50–60%), ovary (20–30%), cervix (5–15%), myometrium (2.5%), and vagina/vulva (1%)^8,9^. Tubal TB is the main cause of FGTB infertility. FGTB can block one or both fallopian tubes, leading to loss of function and further affecting the fertilization process^10,11^. FGTB can also promote endometrial vascularization, atrophy, and adhesion, damage endometrial receptivity, and affect embryo implantation^12^. In addition, FGTB can affect ovarian reserve function^13^. Patients with FGTB are usually characterized by endocrine disorders, oocyte defects, poor embryo quality, low pregnancy rates, and high miscarriage rates; even after receiving comprehensive treatment, their pregnancy and birth rates are extremely low^14^. Infertility caused by FGTB lasts longer than infertility due to other causes^15^.

Similar to FGTB, latent genital TB (LGTB) can cause female infertility by tubal blockage and endometrial receptivity alteration^16^. LGTB is a form of latent TB infection (LTBI), which refers to a state in which the body produces a persistent immune response to Mtb antigen stimulation; no evidence of active tuberculosis in clinical manifestations, bacteriology, or radiology exists^17,18^. The population with LTBI is substantial, accounting for approximately one-quarter of the global population, with no symptoms or signs of the disease but with Mtb present in tissues^17^. Approximately 5–15% of individuals with LTBI have a risk of reactivation into active TB^17^. LGTB incidence is often underestimated owing to its concealed and difficult-to-diagnose nature. In fact, LGTB incidence is quite high in women with infertility, with approximately 35–50% of women with infertility in India diagnosed with LGTB^19^. LGTB can lead to embryo implantation failure and miscarriage by causing endometrial rejection, reduced sub-endometrial blood flow, and microthrombi formation^20^. In addition to the endometrium, LGTB can have adverse effects on the ovarian reserve function in patients^21^, which may also be an important factor leading to infertility in females.

In vitro fertilization and embryo transfer (IVF-ET) is the best treatment for infertility and can be used for patients with FGTB and LGTB with tubal blockage but with an intact or mildly damaged endometrium^22,23^. However, when the ovarian reserve function is impaired, the ovarian response during IVF can become poor, thereby affecting the treatment outcomes of IVF-ET^15,21^. The regulatory mechanisms of FGTB and LGTB affecting ovarian reserve function remain unclear which limits the early treatment of women with impaired ovarian function caused by FGTB and LGTB. Therefore, this study aimed to explore the effects of FGTB and LGTB on ovarian reserve function and response during IVF and metabolic level regulatory mechanisms. The findings are expected to inform improved clinical management and therapeutic approaches for afflicted women.

## Methods

### Study participants

Between September 2021 and December 2022, 19 patients with FGTB and 16 patients with LGTB were recruited from the Reproductive Medicine Center of the Affiliated Hospital of Inner Mongolia Medical University (Hohhot, China). The control group consisted of 16 healthy women. All patients provided written informed consent, and the study protocol was approved by the Ethics Review Committee of Inner Mongolia Medical University. The diagnostic criteria for the FGTB group were thickened and rigid fallopian tubes with caseous tissue and confirmed TB nodules and patients undergoing anti-TB treatment^5^. The diagnostic criteria for the LGTB group were positive serum TB-interferon-gamma release assay, clear pelvic inflammation observed under laparoscopy, and exclusion of other common pathogenic bacterial infections without active TB symptoms^24^.

The inclusion criteria were women aged 20–35 years with primary or secondary infertility for > 2 years and regular menstrual cycles. Women who underwent their first IVF-ET treatment and controlled ovarian hyperstimulation (COH) using an antagonist protocol were also included. The exclusion criteria were women younger or older than 20 or 35 years, respectively, previous ovarian surgery, and a history of endocrine diseases, such as polycystic ovarian syndrome (PCOS). Women who had taken hormones or reproductive toxic drugs in the past 6 months were also excluded.

### Antagonist protocol for ovulation induction

After a qualified examination on the 2nd or 3rd day of the menstrual cycle, an antagonist protocol for ovulation induction was initiated. Gonadotropin (Gn) (Merck Serono, Geneva, Switzerland) was used to stimulate the ovaries, and vaginal ultrasound and hormone levels were monitored every 2–3 days. When the maximum follicle diameter reached 12–14 mm, 0.25 mg of Gn releasing hormone analogs (Merck Serono, Geneva, Switzerland) was subcutaneously administered daily. When at least two follicles diameter ≥ 18 mm or three follicles diameter ≥ 17 mm appeared, human chorionic Gn (hCG) injection (Livzon Pharmaceutical Group Inc., Zhuhai, China) was used to trigger ovulation. The specific dose of medication was adjusted according to the ovarian response of each patient.

### Collection of basic and clinical information

Basic and clinical information of the included patients were collected, including age, duration of infertility, body mass index (BMI), basal follicle-stimulating hormone (FSH), anti-Müllerian hormone (AMH), antral follicular count (AFC), Gn days, Gn dosage, estradiol (E2) level on the trigger day upon hCG administration, and number of oocytes retrieved.

### Sample collection and preprocessing

Oocytes were retrieved from the patients 36 h after hCG injection. The remaining follicular fluid after oocyte retrieval was mixed, centrifuged at 400 × *g* for 15 min using a Centrifuge 5424 R (Eppendorf, Hamburg, Germany), and the supernatant was transferred to a new labeled centrifuge tube and stored at − 80 °C.

The follicular fluid samples were thawed at 4 °C, and 200 µL of the sample was transferred to a 1.5 mL centrifuge tube. Then, 400 µL of methanol (CAS: 67-56-1, Fisher Chemical, Massachusetts, USA) /acetonitrile (CAS: 75-05-8, Fisher Chemical, Massachusetts, USA) (1:1, v/v) was added for metabolite extraction. After vortexing for 30 s, the mixture was extracted using low temperature ultrasound for 30 min (5 °C, 40 kHz). After allowing the mixture to stand at − 20 °C for 30 min, it was centrifuged at 13,000 × *g* for 15 min. The supernatant was dried in a vacuum centrifuge and re-dissolved with 100 μL of acetonitrile/water (1:1, v/v) for subsequent liquid chromatography–mass spectrometry (LC–MS) analysis. To monitor the stability and repeatability of the instrument analysis, 20 µL of the supernatant from each sample was mixed to obtain a quality control (QC) sample, which was analyzed together with the other samples.

### LC–MS analysis

The LC–MS analysis of the samples was conducted on a Thermo UHPLC-Q Exactive HF-X system (Thermo Scientific, Massachusetts, USA) equipped with an ACQUITY HSS T3 column (100 × 2.1 mm i.d., 1.8 μm) at Majorbio Bio-Pharm Technology Co. Ltd. (Shanghai, China). Mobile phase A consisted of 95% water and 5% acetonitrile (containing 0.1% formic acid), whereas mobile phase B consisted of 47.5% acetonitrile, 47.5% isopropanol, and 5% water (containing 0.1% formic acid). The flow rate was 0.40 mL/min, column temperature was 40 °C, and injection volume was 3 μL. The samples were analyzed using electrospray ionization mass spectrometry in positive and negative ion scanning modes. The scan range was 70–1050 m/z; sheath gas flow rate was 50 psi; auxiliary gas flow rate was 13 psi; auxiliary gas heating temperature was 425 °C; ion transfer tube temperature was 325 °C; spray voltage was -3.5 kV and 3.5 kV in negative and positive ion modes, respectively; and the collision energy was set to 20, 40, and 60 eV. The first-stage mass resolution was 60,000, and the second-stage mass resolution was 7,500. Data acquisition was performed in the DDA mode.

### Data analysis of LC–MS

The raw LC–MS data were imported into Progenesis QI (Waters Corporation, Milford, USA) for data processing. A data matrix containing the retention time, m/z, and peak intensity information was obtained. Metabolite identification was performed using the HMDB (http://www.hmdb.ca/), Metlin (https://metlin.scripps.edu/), and Majorbio databases.

The data matrix obtained by searching the database was uploaded to the Majorbio Cloud platform (https://cloud.majorbio.com) for analysis. To obtain a normalized data matrix, the data matrix was first preprocessed, and the response intensities of the sample spectral peaks were normalized using the total sum normalization method. Variables with a relative standard deviation (RSD) > 30% in the QC samples were removed, and the data matrix was log10 transformed to obtain the final data matrix for subsequent analysis.

The processed data matrix was subjected to principal component analysis (PCA) and orthogonal projections to latent structure discriminant analysis (OPLS-DA), followed by a seven-cycle interactive validation to assess the stability of the models. PCA is an unsupervised multivariate statistical analysis method that reflects the overall differences between sample groups and the variability within samples. OPLS-DA is a supervised multivariate statistical discriminant analysis method that removes irrelevant differences to select the differential variables. Partial least-square regression was used to establish a relationship model between metabolite expression levels and sample categories, allowing for sample category prediction. Significantly different metabolites were determined based on the variable importance of projection (VIP) values obtained from the OPLS-DA model (VIP > 1) and the P-values from Student's t-test (P < 0.05).

Differential metabolites were annotated and subjected to pathway enrichment analysis using the Kyoto Encyclopedia of Genes and Genomes (KEGG) database (https://www.kegg.jp/kegg/ pathway.html) to identify the metabolic pathways involved.

### Statistical analysis

The basic and clinical data previously collected from the included patients were analyzed using IBM SPSS Statistics for Windows, Version 27.0 (IBM Corp, New York, USA). As the data were not normally distributed, they are presented as medians (M) and interquartile ranges (Q). For comparisons between two groups, the Mann–Whitney U test was employed, while the Kruskal–Wallis test was used for comparisons among three or more groups. Statistical significance was established at P < 0.05. The Mann–Whitney U test, a non-parametric method, assesses the medians of two independent samples without the assumption of a normal distribution, making it ideal for datasets with skewed distributions or smaller sample sizes. Similarly, the Kruskal–Wallis test, also non-parametric, determines whether the medians of three or more independent samples are equivalent. It is particularly well-suited for non-normally distributed data or small datasets.

For other comparisons made in this study, we utilized the two-sample t-test with equal variances assumed. Statistical significance was defined at P < 0.05. The degrees of freedom for each t-test were calculated based on the sample sizes of the groups involved in the comparison. Specifically, the degrees of freedom were determined using the formula: df = n1 + n2 − 2, where n1 and n2 are the sample sizes of the two groups being compared. To address the issue of multiple comparisons, we applied the Bonferroni correction method.

In addition to the P-value, we have included effect size measures to enhance the interpretability of our results. We reported Hedges' g as a standardized measure of the mean difference between groups. Hedges' g is a statistic that measures the effect size of the mean difference while correcting for small sample bias. It is calculated by dividing the difference between the means of the two groups by the pooled standard deviation, adjusted for sample size. Hedges' g value (H value) can be interpreted as small (H ≈ 0.2), medium (H ≈ 0.5), or large (H ≈ 0.8) effect sizes. We reported Hedges' g alongside the corresponding P-value to provide a comprehensive understanding of the statistical significance and practical importance of the observed differences.

### Ethics approval and consent to participate

This study was approved by the Ethics Review Committee of Inner Mongolia Medical University, Hohhot, China. All procedures involving human participants were performed in accordance with the ethical standards of institutional and national research committees. Informed consent was obtained from all patients included in the study.

## Results

### Clinical analysis of FGTB and LGTB effects on ovarian reserve function

In this study, 51 women undergoing assisted reproduction were recruited, including 19 patients with FGTB (FGTB group), 16 patients with LGTB (LGTB group), and 16 healthy controls (control group). A comparison of basic and clinical information revealed no significant differences in age, BMI, or duration of infertility among the control, FGTB, and LGTB groups (P > 0.05, H ≈ 0.2). However, significant differences were observed in AMH, basal FSH, AFC, Gn days, Gn dosage, E2 on the trigger day upon hCG administration, and the number of retrieved oocytes (P < 0.05, H > 0.8). Levels of robust markers of ovarian reserve, including AMH, AFC, and the number of retrieved oocytes, in the FGTB and LGTB groups were lower than those in the control group, and the basal FSH level was higher in the FGTB and LGTB groups than in the control group. However, during IVF, levels of ovarian response markers, including Gn days and dosage, in the FGTB and LGTB groups were higher than those in the control group, and E2 level on the trigger day upon hCG administration in the FGTB and LGTB groups was lower than that in the control group (Table 1).

**Table 1:** Comparison of basic and clinical information for the FGTB, LGTB, and control groups.

Characteristic	FGTB group (n = 19)	LGTB group (n = 16)	Control group (n = 16)	H value	P value
Age (years)	32 (30.5, 33)*	31.5 (30, 33)*	32.5 (29.8, 33.3)	0.27	0.88
BMI (kg/m^2^)	21.9 (21.7, 22.8)*	22.39 (21.6, 22.7)*	22.38 (21.8, 22.7)	0.03	0.99
Infertility (years)	4 (3, 4)*	3.5 (3, 4.3)*	4 (2.8, 4.3)	0.12	0.94
AMH (μg/L)	0.82 (0.6, 1.1)↓	1.57 (1.3, 1.8)↓	3.29 (2.9, 3.5)^a^	17.56	< 0.001
Basal FSH (mIU/mL)	8.9 (7.8, 10.5)↑	7.065 (6.7, 8.2)↑	5.535 (4.7, 6)^ab^	15.16	< 0.001
AFC	6 (5.5, 9.5)↓	10.5 (7, 12.3)↓	17 (14.5, 18)^ab^	15.32	< 0.001
Gn days	12 (10.5, 12.5)↑	11 (10.8, 11.3)↑	10 (8.8, 10)^ab^	10.38	< 0.001
Gn dosage (U)	3300 (3075, 3637.5)↑	3037.5 (2700, 3225)↑	2531.25 (2337.5, 2943.8)^a^	11.36	< 0.001
E2 (pg/mL)	1628 (869.3, 2110)↓	2539.5 (1691.3, 3967)↓	5110.5 (4902.8, 5484.5)^ab^	13.89	< 0.001
Number of oocytes retrieved	3(2, 5)↓	8 (4, 8.3)↓	14.5 (11.5, 15.3)^ab^	15.30	< 0.001

*FGTB group* patients with female genital tuberculosis, *LGTB group* patients with latent genital tuberculosis, *Control group* healthy controls, *BMI* body mass index, *AMH* anti-Müllerian hormone, *Basal FSH* basal follicle-stimulating hormone, *AFC* antral follicular count, *Gn* gonadotropin; *E2* estradiol on trigger day upon human chorionic gonadotropin (hCG) administration.

*No clear difference was found compared to the control group;.

↓Indicates a lower level compared to the control group.

↑Indicates a higher level compared to the control group.

^a^Statistically significant difference between the control and FGTB groups (P < 0.05, calculated by Mann–Whitney U test).

^b^Statistically significant difference between the control and LGTB groups (P < 0.05, calculated by Mann–Whitney U test); H value was calculated by Hedges’ g, a statistic that measures the effect size of the mean difference while correcting for small sample bias. H value can be interpreted as small (H ≈ 0.2), medium (H ≈ 0.5), or large (H ≈ 0.8) effect sizes; P value in the last column of the table was calculated by Kruskal‒Wallis test, indicating statistical significance at P < 0.05.

### Untargeted metabolomics analysis of differential metabolites between the control and FGTB groups as well as between the control and LGTB groups

#### Quality control evaluation of QC samples

**Figure 1:**
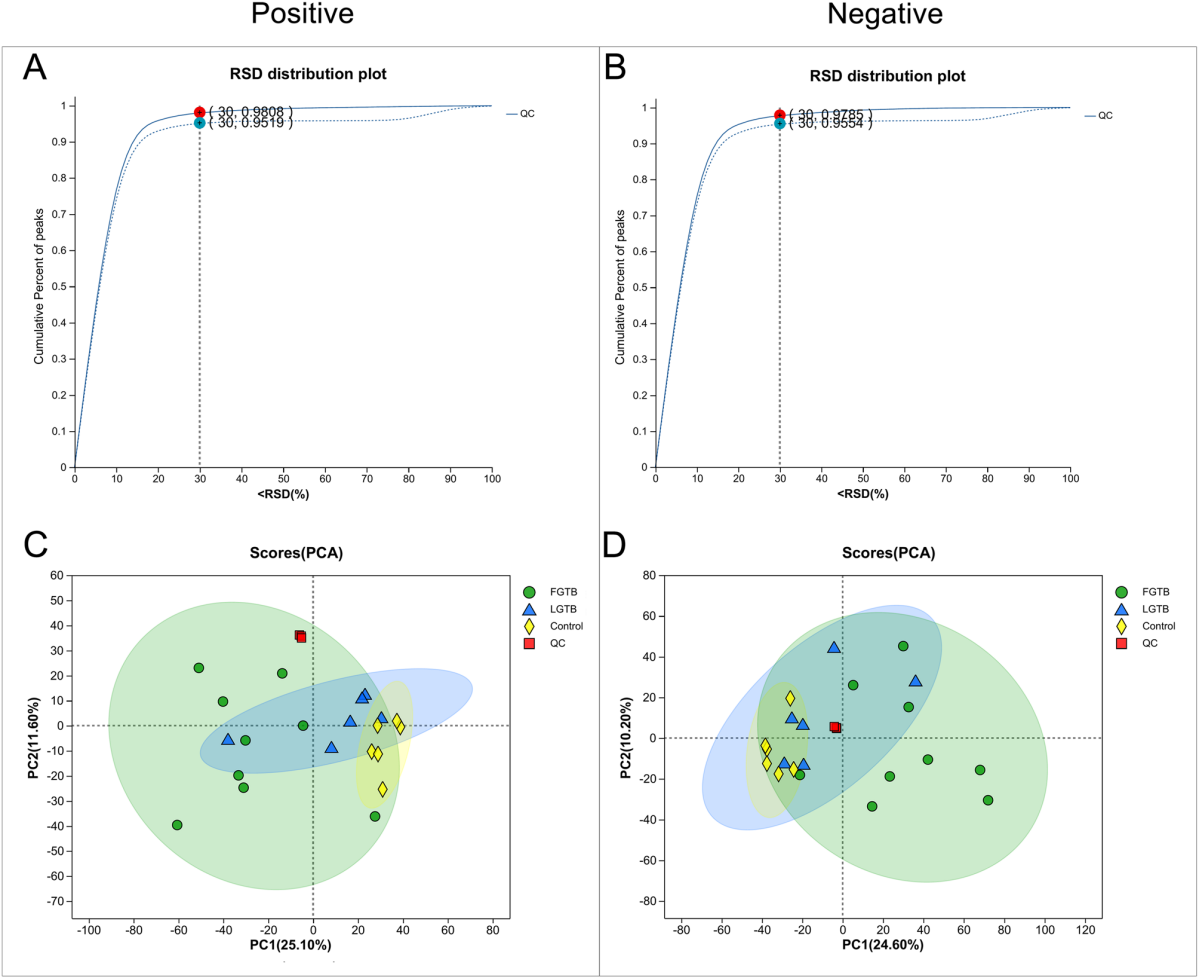
Quality control evaluation of QC samples in the untargeted metabolomics. (**A**) and (**B**) QC sample evaluation charts under positive and negative ion modes, respectively. Dashed lines represent data before preprocessing, while solid lines represent data after preprocessing. After pretreatment, when RSD < 30% in positive ion mode, the cumulative peak proportion was 0.9808, and when RSD < 30% in negative ion mode, the cumulative peak proportion was 0.9785. (**C**) and (**D**) PCA score charts of QC samples under positive and negative ion modes, respectively. After the samples were subjected to dimensionality reduction analysis, they had relative coordinate points on the principal components p1 and p2. The distance between each coordinate point represents the degree of aggregation and dispersion among the samples; a closer distance indicates higher similarity between samples, while a greater distance indicates greater differences between samples. In the figure, the QC samples (red squares) were closely clustered together under both positive and negative ion modes.

To explore the effects of FGTB and LGTB on ovarian reserve function, we performed untargeted metabolomic profiling of follicular fluid in the control, FGTB, and LGTB groups. After data preprocessing, 592 and 480 metabolites were identified in positive and negative ion modes, respectively. The quality of the data was evaluated based on an analysis of the QC samples. For the overall data, an RSD < 0.3 and a cumulative peak proportion > 70% were considered acceptable. QC sample evaluation showed that in both positive and negative ion modes, the data met the acceptance criteria with an RSD < 0.3 and a cumulative peak proportion > 90%, indicating good data quality (Fig. 1A and B). PCA demonstrated that all QC samples (indicated by red squares) clustered closely together, indicating good quality control and reliable data (Fig. 1C and D).

#### Differential analysis of metabolic expression profiles and differential metabolite screening

**Figure 2:**
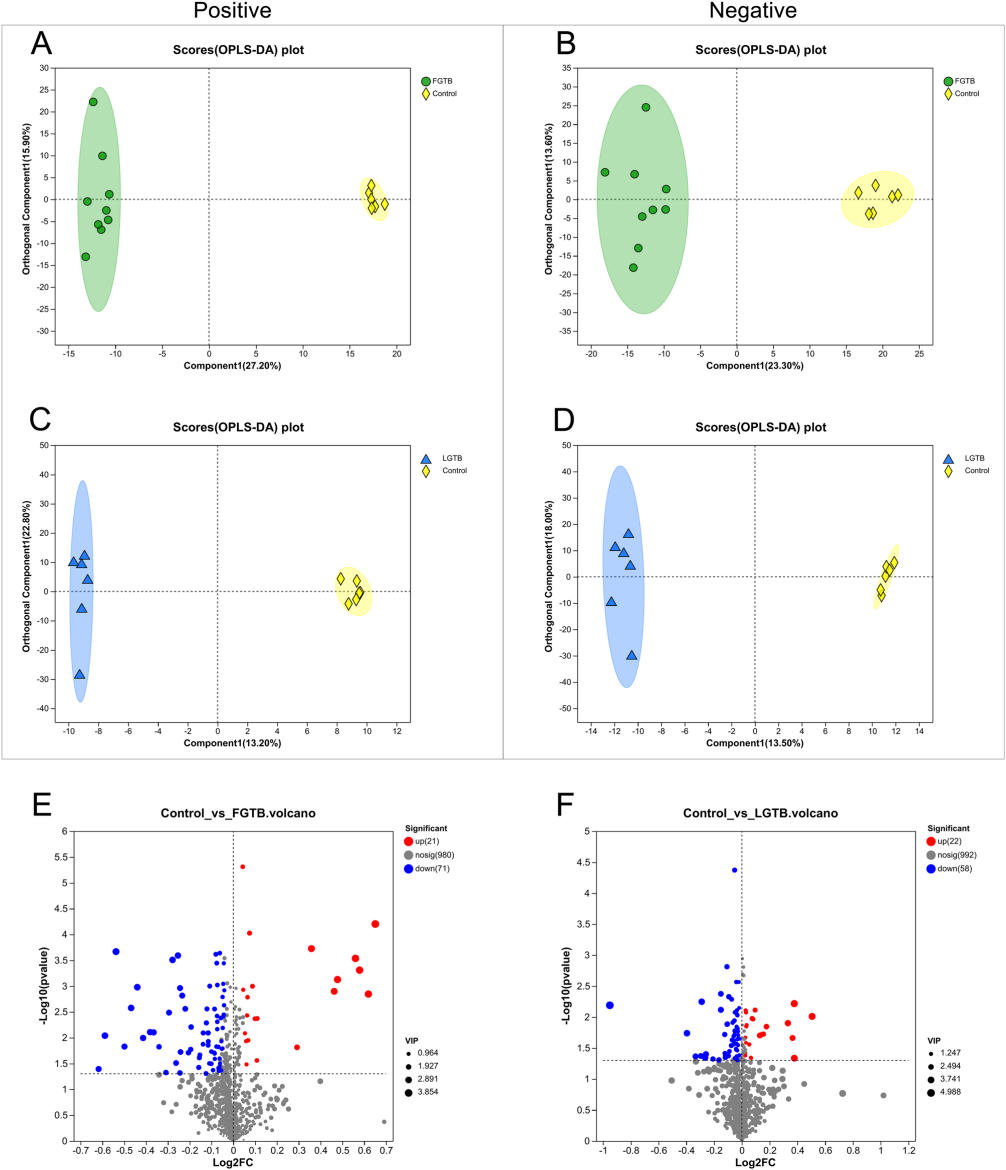
Differential analysis of metabolic expression profiles and differential metabolite screening. (**A**) and (**B**) OPLS-DA score charts of the control and FGTB groups in positive and negative ion modes. The control (yellow diamonds) and FGTB groups (green circles) showed clear separation under both positive and negative ion modes. (**C**) and (**D**) OPLS-DA score charts of the control and LGTB groups in positive and negative ion modes. The control (yellow diamonds) and LGTB groups (blue triangles) showed clear separation under both positive and negative ion modes. (**E**) Differential volcano plot of the control vs. FGTB group. Under the screening criteria of VIP (based on OPLS-DA) > 1 and P < 0.05, 92 differential metabolites were identified in the control vs. FGTB group, with 21 upregulated (red dots) and 71 downregulated (blue dots). (**F**) Differential volcano plot of the control vs. LGTB group. Under the screening criteria of VIP (based on OPLS-DA) > 1 and P < 0.05, 80 differential metabolites were identified in the control vs. LGTB group, with 22 upregulated (red dots) and 58 downregulated (blue dots).

After confirming the quality of the data, we performed OPLS-DA to determine whether substantial differences were observed between the control and FGTB groups and control and LGTB groups. OPLS-DA revealed clear separation trends between the control and FGTB groups (Fig. 2A and B) and control and LGTB groups (Fig. 2C and D) in both positive and negative ion modes, thus indicating substantial differences. Subsequently, we used a screening criterion of VIP (based on OPLS-DA) > 1 and P < 0.05 to identify differential metabolites between the control and FGTB groups and control and LGTB groups. Finally, we screened 92 and 80 differential metabolites in the control vs. FGTB and control vs. LGTB groups, respectively. Further analysis based on volcano plots revealed that among the 92 differential metabolites in the control vs. FGTB group, the levels of 21 were upregulated and 71 were downregulated (Fig. 2E), whereas among the 80 differential metabolites in the control vs. LGTB group, the levels of 22 were upregulated and 58 were downregulated (Fig. 2F).

#### Classification of differential metabolites

**Figure 3:**
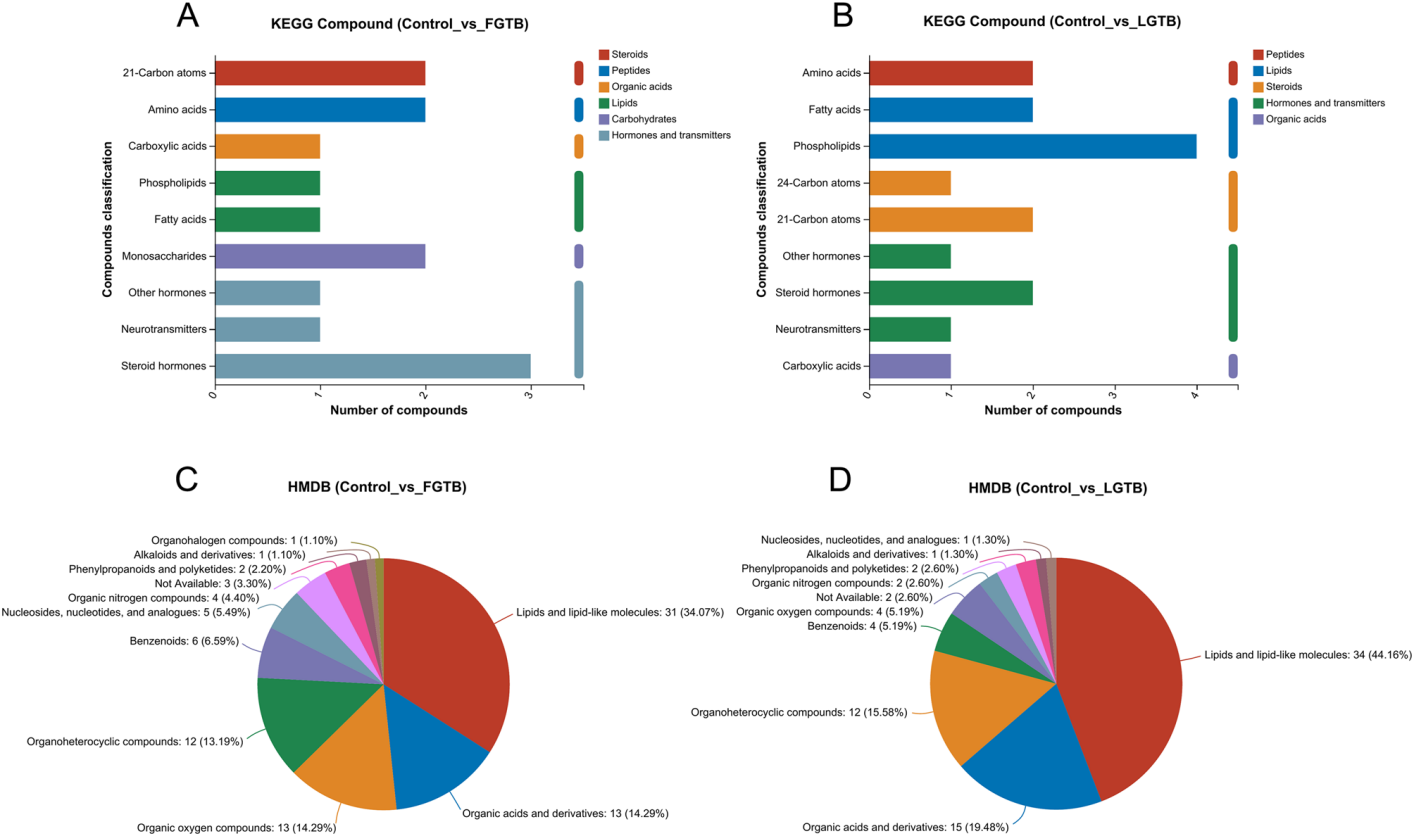
Classification of differential metabolites. (**A**) KEGG compound classification statistics of differential metabolites in the control vs. FGTB group. Among the 92 differential metabolites identified in the control vs. FGTB comparison, two, two, one, two, two, and five differential metabolites were annotated to “Steroids,” “Peptides,” “Organic acids,” “Lipids,” “Carbohydrates,” and “Hormones and transmitters,” respectively, with the remaining metabolites not receiving any annotation. (**B**) KEGG compound classification statistics of differential metabolites in the control vs. LGTB group. Among the 80 differential metabolites identified in the control vs. LGTB comparison, two, six, three, four, and one differential metabolites were annotated to “Peptides,” “Lipids,” “Steroids,” “Hormones and transmitters,” and “Organic acids,” respectively, with the remaining metabolites not receiving any annotation. (**C**) HMDB compound classification statistics of differential metabolites in the control vs. FGTB group. The control vs. FGTB group had 91 out of 92 differential metabolites identified by the HMDB 4.0 database. The 91 differential metabolites were primarily annotated to “Lipids and lipid-like molecules” (31, 34.07%), “Organic acids and derivatives” (13, 14.29%), “Organic oxygen compounds” (13, 14.29%), and “Organoheterocyclic compounds” (12, 13.19%). (**D**) HMDB compound classification statistics of differential metabolites in the control vs. LGTB group. The control vs. LGTB group had 77 out of 80 differential metabolites identified by the HMDB 4.0 database. The 77 differential metabolites were primarily annotated to “Lipids and lipid-like molecules” (34, 44.16%), “Organic acids and derivatives” (15, 19.48%), and “Organoheterocyclic compounds” (12, 15.58%).

We compared the 92 and 80 differential metabolites identified in the control vs. FGTB group and control vs. LGTB group, respectively, using the KEGG compound and HMDB 4.0 databases to obtain annotation information for these differential metabolites. The KEGG compound classification results showed that in the control vs. FGTB group, two, two, one, two, two, and five differential metabolites were annotated to “Steroids,” “Peptides,” “Organic acids,” “Lipids,” “Carbohydrates,” and “Hormones and transmitters,” respectively, and the remaining differential metabolites did not receive any annotation (Fig. 3A). In the control vs. LGTB group, two, six, three, four, and one differential metabolites were annotated to “Peptides,” “Lipids,” “Steroids,” “Hormones and transmitters,” and “Organic acids,” respectively, and the remaining differential metabolites did not receive any annotation (Fig. 3B). The HMDB compound classification results showed that in the control vs. FGTB group, the differential metabolites were primarily annotated to “Lipids and lipid-like molecules,” “Organic acids and derivatives,” “Organic oxygen compounds,” and “Organoheterocyclic compounds”(Fig. 3C). In the control vs. LGTB group, the differential metabolites were primarily annotated to “Lipids and lipid-like molecules,” “Organic acids and derivatives,” and “Organoheterocyclic compounds” (Fig. 3D).

#### KEGG enrichment analysis and topological analysis of differential metabolites

**Figure 4:**
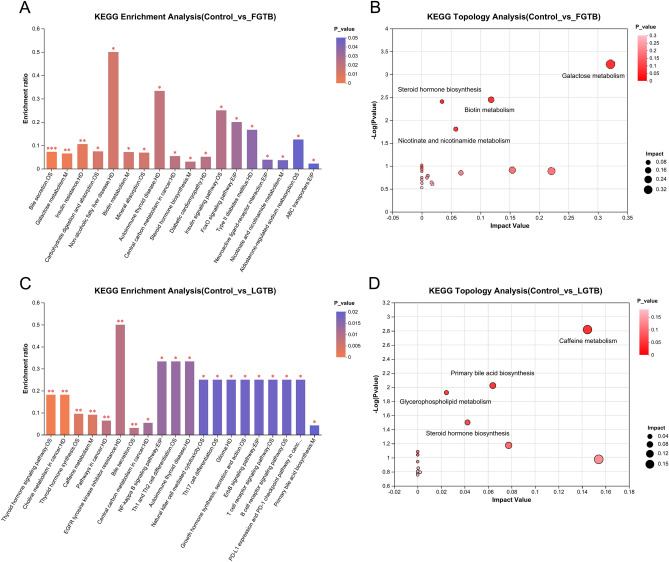
KEGG enrichment analysis and topological analysis. (**A**) KEGG enrichment analysis of differential metabolites in the control vs. FGTB group. There were 18 significantly enriched pathways of differential metabolites in the control vs. FGTB group (P < 0.05), of which four pathways were related to metabolism (M): galactose metabolism, biotin metabolism, steroid hormone biosynthesis, and nicotinate and nicotinamide metabolism. (**B**) KEGG topological analysis of the control vs. FGTB group. The galactose metabolism, biotin metabolism, steroid hormone biosynthesis, and nicotinate and nicotinamide metabolism displayed low P-values (P < 0.05) and high impact values. (**C**) KEGG enrichment analysis of differential metabolites in the control vs. LGTB group. Among the top 20 enriched pathways of differential metabolites in the control vs. LGTB group, only caffeine metabolism and primary bile acid biosynthesis were associated with metabolism (M). (**D**) KEGG topological analysis of the control vs. LGTB group. The caffeine metabolism, primary bile acid biosynthesis, steroid hormone biosynthesis and glycerophospholipid metabolism displayed low P-values (P < 0.05) and high impact values. *OS* Organismal Systems, *M* Metabolism, *HD* Human Diseases, *EIP* Environmental Information Processing. *0.01 < P < 0.05, **0.001 < P < 0.01, ***P < 0.001.

We performed KEGG enrichment and topological analyses of differential metabolites in the control vs. FGTB and control vs. LGTB groups. KEGG enrichment analysis results showed 18 significantly enriched pathways of differential metabolites in the control vs. FGTB group (P < 0.05), of which four pathways were related to metabolism: galactose metabolism, biotin metabolism, steroid hormone biosynthesis, and nicotinate and nicotinamide metabolism (Fig. 4A). KEGG topology analysis revealed that these four metabolic pathways displayed low P-values (P < 0.05) and high impact values between the control and FGTB groups, indicating that these four metabolic pathways were significantly affected (Fig. 4B). Additionally, among the top 20 enriched pathways of differential metabolites in the control vs. LGTB group, only caffeine metabolism and primary bile acid biosynthesis were associated with metabolism (Fig. 4C). KEGG topology analysis revealed that in addition to caffeine metabolism and primary bile acid biosynthesis, steroid hormone biosynthesis and glycerophospholipid metabolism also showed low P-values (P < 0.05) and high impact values, indicating that these four metabolic pathways were significantly affected (Fig. 4D).

### Metabolic level analysis revealed the potential mechanisms of FGTB and LGTB affecting ovarian reserve function

**Figure 5:**
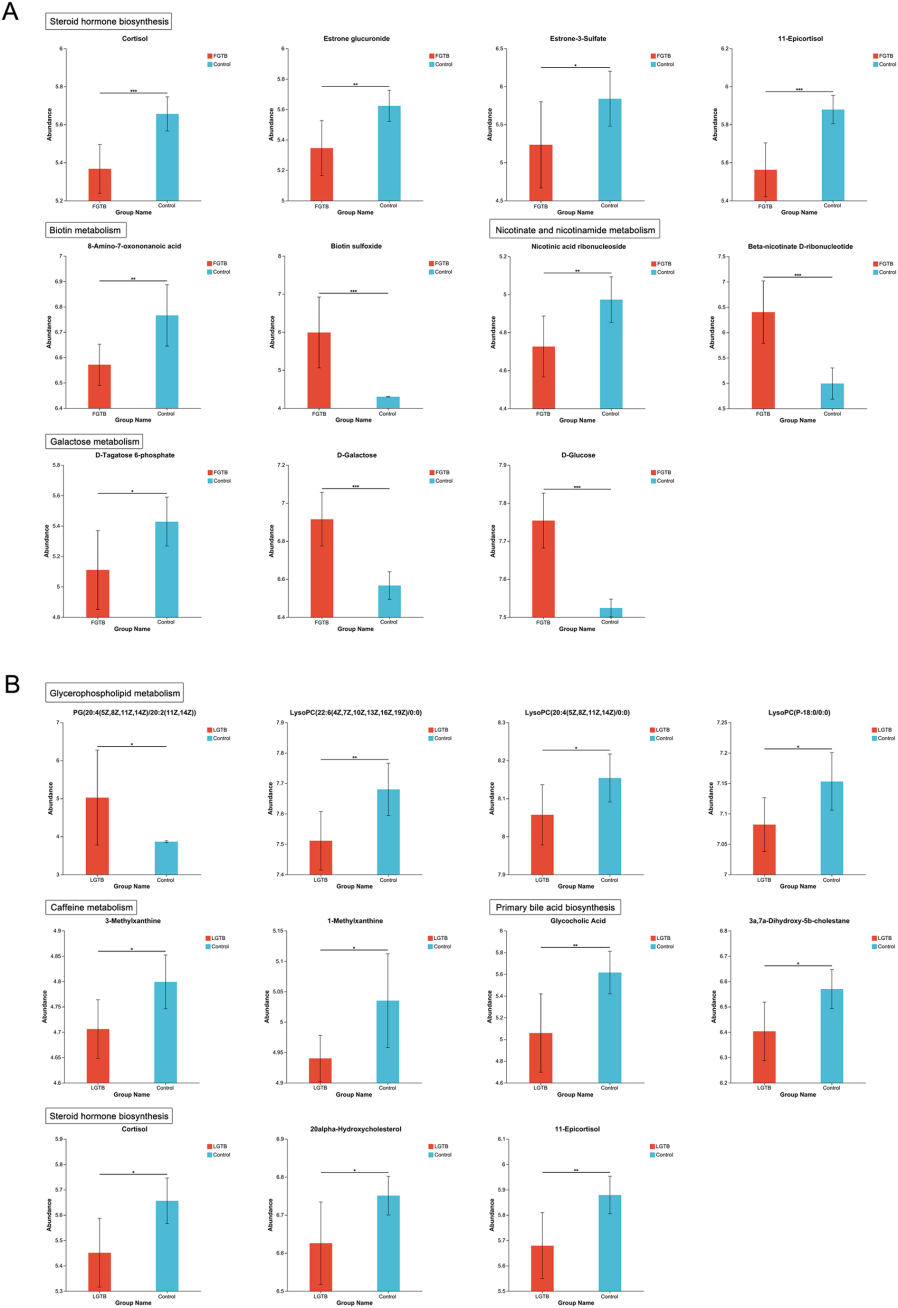
Expression of differential metabolites. (**A**) Expression of differential metabolites in pathways, such as galactose metabolism, biotin metabolism, steroid hormone biosynthesis, and nicotinate and nicotinamide metabolism, in the control vs. FGTB group. Galactose metabolism: D-tagatose 6-phosphate, D-galactose and D-glucose; Biotin metabolism: 8-amino-7-oxononanoic acid and biotin sulfoxide; Steroid hormone biosynthesis: cortisol, estrone glucuronide, estrone-3-sulfate, and 11-epicortisol; Nicotinate and nicotinamide metabolism: nicotinic acid ribonucleoside and beta-nicotinate D-ribonucleotide. (**B**) Expression of differential metabolites in pathways, such as caffeine metabolism, primary bile acid biosynthesis, steroid hormone biosynthesis, and glycerophospholipid metabolism, in the control vs. LGTB group. Caffeine metabolism: 3-methylxanthine and 1-methylxanthine; Primary bile acid biosynthesis: glycocholic acid and 3a, 7a-dihydroxy-5b-cholestane; Steroid hormone biosynthesis: cortisol, 20-alpha-hydroxycholesterol, and 11-epicortisol; Glycerophospholipid metabolism: PG (20:4(5Z,8Z,11Z,14Z)/20:2(11Z,14Z)), lysoPC (22:6(4Z,7Z,10Z,13Z,16Z,19Z)/0:0), lysoPC (20:4(5Z,8Z,11Z,14Z)/0:0), and lysoPC (P-18:0/0:0). *0.01 < P < 0.05, **0.001 < P < 0.01, ***P < 0.001.

KEGG enrichment and topology analyses showed significant changes (P < 0.05) in galactose metabolism, biotin metabolism, steroid hormone biosynthesis, and nicotinate and nicotinamide metabolism in the control vs. FGTB group, and caffeine metabolism, primary bile acid biosynthesis, steroid hormone biosynthesis, and glycerophospholipid metabolism in the control vs. LGTB group. These metabolic pathway changes may be important for the effects of FGTB and LGTB on ovarian reserve function. Further analysis showed that compared with the control group, the FGTB group exhibited D-tagatose 6-phosphate expression downregulation and D-galactose and D-glucose expression upregulation in galactose metabolism; 8-amino-7-oxononanoic acid expression downregulation and biotin sulfoxide expression upregulation in biotin metabolism; cortisol, estrone glucuronide, estrone-3-sulfate, and 11-epicortisol expression downregulation in steroid hormone biosynthesis; and nicotinic acid ribonucleoside expression downregulation and beta-nicotinate D-ribonucleotide expression upregulation in nicotinate and nicotinamide metabolism (Table 2 and Fig. 5A). Additionally, the LGTB group showed 3-methylxanthine and 1-methylxanthine expression downregulation in caffeine metabolism; glycocholic acid and 3a, 7a-dihydroxy-5b-cholestane expression downregulation in primary bile acid biosynthesis; cortisol, 20-alpha-hydroxycholesterol, and 11-epicortisol expression downregulation in steroid hormone biosynthesis; and PG (20:4(5Z,8Z,11Z,14Z)/20:2(11Z,14Z)) expression upregulation and lysoPC (22:6(4Z,7Z,10Z,13Z,16Z,19Z)/0:0), lysoPC (20:4(5Z,8Z,11Z,14Z)/0:0), and lysoPC (P-18:0/0:0) expression downregulation in glycerophospholipid metabolism (Table 3 and Fig. 5B).

**Table 2:** Differential metabolites in galactose metabolism, biotin metabolism, steroid hormone biosynthesis, and nicotinate and nicotinamide metabolism of the control vs. FGTB group.

Metabolites	Expression	VIP(OPLS-DA)	P value	FC	Pathways
Nicotinic acid ribonucleoside	Decreased	1.2019	0.006841	0.9503	Nicotinate and nicotinamide metabolism
Cortisol	Decreased	1.4356	0.0003595	0.9489	Steroid hormone biosynthesis
8-Amino-7-oxononanoic acid	Decreased	1.1293	0.002357	0.9712	Biotin metabolism
Biotin sulfoxide	Increased	3.3614	0.0007475	1.3928	Biotin metabolism
Beta-nicotinate D-ribonucleotide	Increased	3.1369	0.0001883	1.282	Nicotinate and nicotinamide metabolism
D-Tagatose 6-phosphate	Decreased	1.2795	0.01957	0.9414	Galactose metabolism
Estrone glucuronide	Decreased	1.3115	0.004916	0.9506	Steroid hormone biosynthesis
Estrone-3-Sulfate	Decreased	1.6477	0.0377	0.8962	Steroid hormone biosynthesis
11-Epicortisol	Decreased	1.4826	0.0002425	0.9461	Steroid hormone biosynthesis
D-Galactose	Increased	1.5562	9.45E-05	1.0532	Galactose metabolism
D-Glucose	Increased	1.3186	4.89E-06	1.0306	Galactose metabolism

The variable importance of projection (VIP) value was obtained from the OPLS-DA analysis. Generally, metabolites with VIP ≥ 1 are considered to have significant differences. The P value was obtained from the Student's t-test, and metabolites with P value < 0.05 are generally considered to have significant differences. Fold Change (FC) was obtained from univariate analysis.

**Table 3:** Differential metabolites in caffeine metabolism, primary bile acid biosynthesis, steroid hormone biosynthesis, and glycerophospholipid metabolism of the control vs. LGTB group.

Metabolites	Expression	VIP(OPLS-DA)	P value	FC	Pathways
LysoPC (22:6(4Z,7Z,10Z,13Z,16Z,19Z)/0:0)	Decreased	1.6412	0.009328	0.978	Glycerophospholipid metabolism
LysoPC (20:4(5Z,8Z,11Z,14Z)/0:0)	Decreased	1.1411	0.04129	0.9881	Glycerophospholipid metabolism
Cortisol	Decreased	1.7534	0.01143	0.9638	Steroid hormone biosynthesis
PG (20:4(5Z,8Z,11Z,14Z)/20:2(11Z,14Z))	Increased	3.8661	0.04618	1.3	Glycerophospholipid metabolism
LysoPC (P-18:0/0:0)	Decreased	1.0213	0.02249	0.9901	Glycerophospholipid metabolism
Glycocholic Acid	Decreased	3.0297	0.007655	0.9008	Primary bile acid biosynthesis
20alpha-Hydroxycholesterol	Decreased	1.188	0.02849	0.9815	Steroid hormone biosynthesis
3a, 7a-Dihydroxy-5b-cholestane	Decreased	1.4133	0.01437	0.9746	Primary bile acid biosynthesis
3-Methylxanthine	Decreased	1.0337	0.01536	0.9806	Caffeine metabolism
1-Methylxanthine	Decreased	1.0214	0.02194	0.9811	Caffeine metabolism
11-Epicortisol	Decreased	1.5712	0.008384	0.966	Steroid hormone biosynthesis

The variable importance of projection (VIP) value was obtained from the OPLS-DA analysis. Generally, metabolites with VIP ≥ 1 are considered to have significant differences. The P value was obtained from the Student's t-test, and metabolites with P value < 0.05 are generally considered to have significant differences. Fold Change (FC) was obtained from univariate analysis.

## Discussion

TB, a global health concern, is known to affect various organ systems, but its effects on fertility have been less well-documented^25,26^. Ovarian reserve function refers to the ability of the growth and development of follicles and oocyte fertilization after ovulation, which is determined by the quantity and quality of the remaining oocytes in the ovaries and reflects a woman's reproductive potential^27^. The most commonly used biomarkers in clinical practice for assessing the ovarian reserve function include age, basal FSH, AFC, and AMH^28^. This study provided clear evidence that FGTB and LGTB have detrimental effects on ovarian reserve, as indicated by significantly reduced AMH levels, decreased AFC counts, elevated basal FSH levels, and fewer retrieved oocytes in afflicted women compared to controls (P < 0.05). In the field of reproductive medicine, the findings of this study have made an important contribution to our understanding of the impact of FGTB and LGTB on female fertility. Additionally, the study also found that FGTB and LGTB can affect the ovarian response of afflicted women during IVF-ET treatment. Ovarian response refers to the sensitivity of the ovaries to exogenous Gn during COH^29^. The ovarian response determines whether a suitable number of oocytes can be recruited, which is one of the factors for the success of COH and directly affects the entire ovulation induction process and outcome of assisted reproduction^30^. In this study, the E2 level after hCG administration and the number of oocytes retrieved in the FGTB and LGTB groups were significantly lower than those in the control group (P < 0.05), whereas the Gn days and doses were higher than those in the control group. This indicates that the ovarian response decreases during IVF-ET treatment in patients with FGTB and LGTB, which is not beneficial for these patients^31^. This study has made an important contribution to our understanding of the impact of FGTB and LGTB on ovarian reserve function and ovarian response during IVF. These findings highlight the need for further research and the development of targeted therapeutic strategies to address the fertility challenges faced by afflicted women.

The use of untargeted metabolomics to analyze follicular fluid provides a novel approach to reveal the potential mechanisms by which FGTB and LGTB affect ovarian function. Follicular fluid provides a microenvironment for oocyte growth and development, and its metabolites support oocyte development and directly affect oocyte quality^32^. In this study, we identified 92 and 80 differential metabolites in the FGTB and LGTB groups, respectively, through untargeted metabolomics, indicating complex metabolic changes in the follicular fluid of patients with FGTB and LGTB. The pathway enrichment analysis and topology analysis further elucidated specific metabolic pathways that were significantly altered (P < 0.05), such as galactose metabolism, biotin metabolism, steroid hormone biosynthesis, and nicotinate and nicotinamide metabolism in the FGTB group, and caffeine metabolism, primary bile acid biosynthesis, steroid hormone biosynthesis, and glycerophospholipid metabolism in the LGTB group, providing a clearer picture of the biochemical changes that contribute to ovarian dysfunction. In this study, D-galactose and D-glucose expression levels in the galactose metabolism of the FGTB group were upregulated, and the accumulation of these metabolites may hinder the development of oocytes, and induce oxidative stress and other adverse reactions, thereby damaging ovarian reserve function^33,34^. Steroid hormones play key regulatory roles in follicular development^35–38^. In this study, cortisol and estrone glucuronide levels were downregulated in the steroid hormone biosynthesis of the FGTB group, and the reduced secretion of these metabolites may affect oogenesis and follicle development, leading to a decline in ovarian reserve function^35,37^. The effects of biotin metabolism, as well as nicotinate and nicotinamide metabolism, on oogenesis and ovarian reserve function have not been reported and warrant further investigation. Additionally, bile acid metabolites and the bile acid receptor farnesoid X receptor (FXR) play important roles in follicular development and ovarian function^39–41^. Significant changes in primary bile acid biosynthesis were observed in the LGTB group (P < 0.05), with downregulation of glycocholic acid and 3a,7a-dihydroxy-5b-cholanate levels, suggesting that LGTB may affect ovarian reserve function by regulating primary bile acid biosynthesis and reducing the secretion of bile acid metabolites. Similar to FGTB, LGTB may also affect follicular development and oocyte maturation by regulating the steroid hormone biosynthesis pathway and reducing cortisol secretion. Lysophosphatidylcholines (lysoPCs) in glycerophospholipids can activate extracellular signal-regulated kinases and protein kinase C, which are involved in the regulation of follicular development and oocyte maturation^42^. The downregulation of lysoPCs in glycerophospholipid metabolism in the LGTB group may also be one of the reasons for the decline in ovarian reserve function. Furthermore, the levels of 3-methylxanthine and 1-methylxanthine in the caffeine metabolism were downregulated in the LGTB group, but the effects of these metabolites on follicular development and ovarian reserve function are not yet clear. These findings fill a critical gap in the current understanding of genital tuberculosis and its reproductive consequences. Previous studies have indicated a connection between tuberculosis and infertility^43^, but the specific mechanisms have remained elusive. The metabolomic insights from this study offer a more detailed understanding of how tuberculosis may disrupt ovarian function, potentially contributing to infertility.

The findings of our study provide novel insights into the metabolic mechanisms underlying the regulation of ovarian reserve function by FGTB and LGTB. These insights have considerable implications for clinical practice and offer potential avenues for improving the diagnosis and treatment of women with these conditions. Our understanding of the metabolic alterations associated with FGTB and LGTB could pave the way for the development of new diagnostic markers or panels. These could allow for earlier detection and more precise characterization of these conditions, leading to timely interventions and improved outcomes. For example, identifying specific metabolites or metabolic pathways that are dysregulated in FGTB and LGTB could serve as biomarkers to distinguish these conditions from other causes of ovarian dysfunction^44^. The specific metabolic pathways identified in our study offer a promising direction for the development of therapeutic interventions. Restoring or modulating metabolic imbalances may be possible to preserve or enhance ovarian reserve function in women with FGTB and LGTB. For instance, if certain metabolites are found to be protective, they could be used as supplements or therapeutics to mitigate the decline in ovarian function^45,46^. Incorporating the metabolic aspects of ovarian reserve function into patient education and counseling is crucial for empowering individuals with FGTB and LGTB to make informed decisions about their reproductive health. By understanding the metabolic underpinnings of their condition, patients can better adhere to treatment plans and adopt lifestyle modifications that support metabolic health. This could improve patient engagement and ultimately lead to better reproductive outcomes^47^.

While our study has contributed to the current knowledge of the metabolic regulation of ovarian reserve function, there is still much to be explored. Future research should focus on validating our findings in larger, diverse populations and investigating the specific mechanisms by which FGTB and LGTB exert their effects at the metabolic level. Furthermore, translational studies are needed to test the feasibility and efficacy of potential therapeutic interventions based on our metabolic insights.

In conclusion, our study found that ovarian reserve function and response during IVF decreased in patients with FGTB and LGTB. Our metabolomics analysis of follicular fluid suggested that FGTB and LGTB may lead to a decline in ovarian reserve function through the regulation of specific metabolic pathways. Our study highlights the importance of considering metabolic changes in the context of FGTB and LGTB's impact on ovarian reserve function. It provides a foundation for future research to develop new therapeutic strategies that may improve reproductive outcomes for women affected by these conditions. By integrating metabolomics analysis into diagnostic and treatment protocols, healthcare providers can offer more personalized and effective care approaches for these patients.

## Data Availability

The datasets used and analyzed during the current study are available from the corresponding author on reasonable request.

## Abbreviations

FGTB: Female genital tuberculosis
LGTB: Latent genital tuberculosis
IVF-ET: In vitro fertilization and embryo transfer
IVF: In vitro fertilization
TB: Tuberculosis
Mtb: *Mycobacterium tuberculosis*
PTB: Pulmonary tuberculosis
EPTB: Extrapulmonary tuberculosis
LTBI: Latent tuberculosis infection
COH: Controlled ovarian hyperstimulation
Gn: Gonadotropin
hCG: Human chorionic gonadotropin
BMI: Body mass index
FSH: Follicle-stimulating hormone
AMH: Anti-mullerian hormone
AFC: Antral follicular count
E2: Estradiol
LC–MS: Liquid chromatography–mass spectrometry
QC: Quality control
RSD: Relative standard deviation
PCA: Principal component analysis
OPLS-DA: Orthogonal projections to latent structures-discriminant analysis
VIP: Variable importance of projection
KEGG: Kyoto encyclopedia of genes and genomes
FXR: Farnesoid X receptor
